# An enhanced nonparametric quality control chart with application related to industrial process

**DOI:** 10.1038/s41598-024-64084-7

**Published:** 2024-06-12

**Authors:** Muhammad Abid, Mei Sun, Aroosa Shabbir, M. E. Bakr, Tahir Abbas

**Affiliations:** 1 School of Mathematical Sciences, Jiangsu University, Zhenjiang, Jiangsu People’s Republic of China; 2 Department of Statistics, Government College University, Faisalabad, 38000 Pakistan; 3 Department of Statistics and Operations Research, College of Science, King Saud University, P.O. Box 2455, 11451 Riyadh, Saudi Arabia; 4 Department of Mathematics, College of Sciences, University of Sharjah, Sharjah, UAE

**Keywords:** Average run length, Control chart, Normality, Double Homogeneously weighted moving average, Nonparametric, Ranked, Wilcoxon signed-rank test, Engineering, Mathematics and computing

## Abstract

In various practical situations, the information about the process distribution is sometimes partially or completely unavailable. In these instances, practitioners prefer to use nonparametric charts as they don’t restrict the assumption of normality or specific distribution. In this current article, a nonparametric double homogeneously weighted moving average control chart based on the Wilcoxon signed-rank statistic is developed for monitoring the location parameter of the process. The run-length profiles of the newly developed chart are obtained by using Monte Carlo simulations. Comparisons are made based on various performance metrics of run-length distribution among proposed and existing nonparametric counterparts charts. The extra quadratic loss is used to evaluate the overall performance of the proposed and existing charts. The newly developed scheme showed comparatively better results than its existing counterparts. For practical implementation of the suggested scheme, the real-world dataset related to the inside diameter of the automobile piston rings is also used.

## Introduction

Statistical process control (SPC) provides different techniques to screen the processes to increase their quality (cf. Montgomery^1^). Each process shows an unnatural variation in its output. To improve the output of the process, these unnatural variations need to be investigated and reduced. To reduce this variation control charts are widely used to monitor the recent position of the processes and to distinguish between unnatural causes of variations and natural causes of variations. Major types of control charts are Shewhart^2^, exponentially weighted moving average (SEWMA) by Roberts^3^, and cumulative sum (CUSUM) by Page^4^ for monitoring the location and scale parameters. Lucas and Saccucci^5^ suggested an improved SEWMA chart utilizing the abilities of Shewhart charts, combined Shewhart-SEWMA control chart. This chart covers a possible range of both larger and smaller shifts. Haq^6^ developed a hybrid SEWMA (HEWMA) control chart by combining two SEWMA statistics. He investigated that the HEWMA chart performs better than CUSUM, SEWMA, and mixed EWMA-CUSUM schemes but its drawback is when smoothing constants become equal the variance of HEWMA control chart turns undefined. Abbas^7^ proposed a homogeneously weighted moving average (SHWMA) scheme to monitor process mean. The performance of these charts with non-normal processes is under consideration. So, to improve the performance of these control charts, different refinements are invented such as parametric and non-parametric control charts.

Most of the work in SPC is focused on parametric control charts in which the distribution of the monitoring processes is known or normal distribution. But in many applications, the distribution of the monitoring processes is usually non-normal or unknown. So, nonparametric (NP) or distribution-free control charts play a vital role to solve the above-mentioned problem. The main fact in nonparametric charts is that the distribution of run-length (RL) for an in-control (IC) process is the same for all continuous distributions which are not true for parametric control charts (cf. Chakraborti et al.^8^) and Qiu^9^). Chakraborti and Graham^10^ highlighted the drawbacks of parametric control charts and preferred nonparametric charts as these are more efficient, simple, robust, and distribution-free. From few decades many researchers have published many articles on the NP setup.

Yang et al.^11^ and Yang and Cheng^12^ suggested the NP SEWMA sign (NPSN-E) and NP CUSUM sign schemes to detect minor deviations in the process location when the underlying distribution is either non‐normal or unknown. Lu^13^ designed an NP double generally weighted moving average chart using a sign test. Riaz and Abbasi^14^ developed an NP double SEWMA scheme based on the sign statistic under arcsine transformation. Haq^15^ suggested the conforming RL based SEWMA sign chart. Ali et al.^16^ proposed an NP SEWMA sign chart under simple and ranked set sampling schemes. Abbas et al.^17^ developed NP progressive sign scheme to monitor individual data. The main disadvantage of the statistic of the sign test is that it lost the information about the magnitude of observation and simply assign a sign to each observation. Due to this fact, the statistical power of the sign test is decreased. So, in this situation, we have preferred to use Wilcoxon signed rank (WSR) test and because WSR test is more efficient than the sign test. Chakraborty et al.^18^ proposed the idea of distribution-free generally weighted moving average chart based on WSR statistic. Raza et al.^19^ designed NP HWMA control charts by integrating the statistics of sign test and WSR test (NPSR-H), respectively. Both distribution-free schemes performed slightly better than their counterparts. There is a wide list of NP control charts in the SPC literature, e.g., Bakir and Reynolds^20^, Amin et al.^21^, Bakir^22,23^, Das and Bhattacharya^24^, Graham et al.^25^, Graham et al.^26^, Mukherjee et al.^27^, Abbas et al.^28^, Castagliola et al.^29^, Godase et al.^30^, and Mahadik and Godase^31^ etc.

Recently, Abid et al.^32^ developed a double HWMA (DHWMA) chart by using the statistic of the HWMA chart twice. Abid et al.^32^ showed that the performance of the DHWMA is relatively better in case of normal distribution than to the non-normal distribution. So, to overcome this problem, we suggest a new NP DHWMA chart by using the statistic of the WSR test and labeled as NPSR-DH. The main advantage of the NPSR-DH chart is that it can be considered for every kind of data without observing its distribution.

The upcoming sections are arranged as follows: In Section "Wilcoxon signed-rank test", we have presented the design structure of existing and proposed charts based on the WSR test. The performance assessment and RL comparisons are provided in Section "Performance evaluation". An industrial application is offered in Section "Industrial application of the NPSR-DH chart". In the end, the conclusion and recommendations are offered in Section "Conclusion and recommendations".

## Wilcoxon signed-rank test

Suppose $V$ is the quality characteristic and its median is the target value (${\alpha }_{o}$), and ${V}_{it}$ represents the *i*th observation in the *t*th sample of size $n>1$ for $i=1, 2, 3, \cdots , n$ and $t=1, 2, 3, \cdots .$ Let ${R}_{it}^{+}$ measures the absolute rank differences $\left|{V}_{it}-{\alpha }_{o}\right|, i=1, 2, 3, \cdots , n$, then the WSR statistics following Graham et al.^25^ is described as:

1 $${WR}_{t}={\sum }_{i=1}^{n}{I}_{it}{R}_{it}^{+} t=1, 2, 3, \cdots \quad \text{where} \quad {I}_{it}=\left\{\begin{array}{c}1 if \left({V}_{it}-{\alpha }_{o}\right)>0\\ 0 if \left({V}_{it}-{\alpha }_{o}\right)=0 \\ -1 if \left({V}_{it}-{\alpha }_{o}\right)<0\end{array}\right.$$

Here, ${WR}_{t}$ is the sum of ${I}_{it}{R}_{it}^{+}$. It is revealed that that the WSR statistic is linearly associated to the Mann–Whitney test statistic ${W}_{n}^{+}$ through $WR=\frac{2{W}_{n}^{+}-n\left(n+1\right)}{2}$ ((cf. Gibbons and Chakraborti^33^). Based on the relationship between $WR and {W}_{n}^{+}, \text{the WSR}$ statistic has a mean and variance as follows:

$$\mu =0\quad \text{and} \quad {\sigma }^{2}=\frac{n\left(n+1\right)\left(2n+1\right)}{6}$$

Also in WSR test, it is assumed that the distribution of differences between paired observations is symmetric. This implies that there should be an equal number of positive and negative differences, and they should be evenly spread out. Violation of this assumption can undermine the validity of the WSR test results.

### Nonparametric SEWMA signed-rank (NPSR-E) control chart

Graham et al.^25^ developed an NPSR-E control chart by accumulating the statistic ${WR}_{t}$ of Shewhart-type SR chart of Bakir^22^. The charting statistic of the NPSR-E control chart is written as based on (1):

2 $${ES}_{t}=\beta {WR}_{t}+\left(1-\beta \right){ES}_{t-1} \quad \text{for} t=1, 2, 3, \cdots$$

where the initial value of the statistic given in (2) is taken as zero $\left({ES}_{o}=0 \right) \text{and }0<\beta \le 1$ is the smoothing parameter of the NPSR-E chart. The upper and lower time-varying limits of the NPSR-E control chart by using the mean and variance of (2) are as follows:

3 $$UCL\backslash LCL=\left\{\pm L\sqrt{\left(\frac{n\left(n+1\right)\left(2n+1\right)}{6}\right)\left(\frac{\beta }{2-\beta }\right)\left(1-{\left(1-\beta \right)}^{2t}\right)}\right. \quad \text{and} \quad CL=0$$

The “steady-state” control limits when $t\to \infty$ are given below:

4 $$UCL\backslash LCL=\left\{\pm L\sqrt{\left(\frac{n\left(n+1\right)\left(2n+1\right)}{6}\right)\left(\frac{\beta }{2-\beta }\right)}\right. \quad \text{and} \quad CL=0$$

If a single point of ${ES}_{t}$ statistic is plotted outside the control limits then the process is considered out of control (OOC).

### Nonparametric SHWMA signed-rank (NPSR-H) control chart

The NPSR-H control chart was introduced by Raza et al.^19^ for monitoring the process location and exhibit early shift detection. The design structure of NPSR-H chart that is based on (1) is described below:

5 $${H}_{t}=\beta {WR}_{t}+\left(1-\beta \right){\overline{WR} }_{t-1 },$$

where ${\overline{WR} }_{t-1}=\frac{{\sum }_{i=1}^{t-1}{WR}_{i}}{t-1}$ the average of first $(t-1)$ samples of $WR$ statistics and the initial value of ${\overline{WR} }_{o}$ is set equals to zero. For obtaining the parameters of the plotting statistic ${H}_{t}$, given in (5) can also be written as below:

6 $${H}_{t}=\beta {WR}_{t}+\frac{\left(1-\beta \right)}{t-1}\sum_{i=1}^{t-1}{WR}_{i}$$

For obtaining the mean of this chart applying expectation on (6):

$${H}_{t}=\beta E\left({WR}_{t}\right)+\frac{\left(1-\beta \right)}{t-1}\sum_{i=1}^{t-1}E\left({WR}_{i}\right)$$

$$E\left({H}_{t}\right)=0$$

We will get the variance of ${H}_{t}$ as follows:

7 $$\begin{aligned} Var\left({H}_{t}\right)&={\beta }^{2}Var\left({WR}_{t}\right)+{\left(\frac{1-\beta }{t-1}\right)}^{2}{\sum }_{i=1}^{t-1}Var\left({WR}_{i}\right) \\ Var\left({H}_{t}\right)&=\left\{\begin{array}{ll}{\beta }^{2}\left(\frac{n\left(n+1\right)\left(2n+1\right)}{6}\right) & \quad \text{for}\,t=1\\ \frac{n\left(n+1\right)\left(2n+1\right)}{6}\left({\beta }^{2}+\frac{{\left(1-\beta \right)}^{2}}{t-1}\right) & \quad \text{for}\,t>1\end{array}\right.\end{aligned}$$

The control limits corresponding to the NPSR-H chart are given below:

8 $$UCL/LCL=\pm T\sqrt{Var\left({H}_{t}\right)}$$

where T denotes the width of the control limits based on the IC ARL and $Var\left({H}_{t}\right)$ is defined in (7). In (7), if the no of samples approaches to infinity then the variance will be ${\beta }^{2}\left(\frac{n\left(n+1\right)\left(2n+1\right)}{6}\right)$. Therefore, the control limits of the NPSR-H chart based on asymptotic variance are given as below (cf. Raza et al.^19^):

9 $${UCL}/{LCL}=\pm T\sqrt{{\beta }^{2}\left[\frac{n\left(n+1\right)\left(2n+1\right)}{6}\right]}$$

### Nonparametric SEWMA sign (NPSN-E) control chart

Yang et al.^11^ suggested the NPSN-E for monitoring of changes in process location. The plotting statistics of NPSN-E chart is defined as:

10 $${E}_{t}=\beta {M}_{t}+\left(1-\beta \right){M}_{t-1}$$

where ${M}_{t}=\sum_{i=1}^{n}{I}_{i}$ and ${I}_{i}=1$ if $\left({V}_{it}-{\alpha }_{o}\right)>0$, otherwise, ${I}_{i}=0$. The starting value of ${E}_{t}$ is the mean of ${M}_{t}$; i.e. ${M}_{o}=E\left({E}_{o}\right)=np$. The mean and variance of the NPSN-E chart are: ${\mu }_{o}=np and {\sigma }_{0}^{2}=\frac{\beta \left[1-{\left(1-\beta \right)}^{2t}\right]}{2-\beta }\left[np\left(1-p\right)\right]$. If the time is infinite then the asymptotic variance NPSN-E chart, ${\sigma }_{0}^{2}=\frac{\beta }{2-\beta }\left[np\left(1-p\right)\right]$.

Therefore, the asymptotic control limits of NPSN-E chart are:

11 $$\begin{array}{l}UCL=np+L\sqrt{\frac{\beta }{2-\beta }\left[np\left(1-p\right)\right]}\\ CL=np \\ LCL=np-L\sqrt{\frac{\beta }{2-\beta }\left[np\left(1-p\right)\right]}\end{array}$$

where $\beta$ and $L$ are set according to the desire ${ARL}_{o}$.

### Parametric SEWMA control chart

Robert^3^ developed SEWMA control chart for detecting of the small shifts in process location. Robert^3^ defined the plotting statistic of SEWMA chart as follows:

12 $${EWMA}_{i}=\beta {\overline{V} }_{i}+(1-\beta ){EWMA}_{i-1}$$

where $\beta$ lies in the range $0<\beta \le 1$. The intial value of ${EWMA}_{0}={\mu }_{o}$. The IC mean and asymptotic variance of the SEWMA chart are: $E\left({EWMA}_{i}\right)={\mu }_{o}$ and $Var\left({EWMA}_{i}\right)=\frac{{\sigma }_{0}^{2}}{n}\left(\frac{\beta }{2-\beta }\right)$. The control limits constructed on the basis of the aforementioned mean and variance for the SEWMA chart is given as:

13 $$\left.\begin{array}{c}LCL={\mu }_{0}-L\frac{{\sigma }_{0}}{\sqrt{n}}\sqrt{\left(\frac{\beta }{2-\beta }\right)},\\ CL={\mu }_{0},\\ UCL={\mu }_{0}+L\frac{{\sigma }_{0}}{\sqrt{n}}\sqrt{\left(\frac{\beta }{2-\beta }\right).}\end{array}\right]$$

where $L$ is the control limits coefficient, which depends on $\beta$ and ${ARL}_{o}$.

### Parametric SHWMA control chart

Abbas^7^ proposed a SHWMA chart that allocates equal weights to the previous observation, unlike the EWMA chart. The plotting statistic of HWMA chart is given as:

14 $${HWMA}_{i}=\beta {\overline{V} }_{i} + (1-\beta ){\overline{\overline{V}}}_{i-1}$$

where ${\overline{\overline{V}}}_{i-1}=\frac{\sum_{i=1}^{t-1}{\overline{Z} }_{i}}{t-1}$ is the mean of the previous $t-1$ samples and the starting value of ${\overline{\overline{Z}}}_{0}={\mu }_{o}.$

The control limits of SHWMA chart based on the statistic (14) are defined as:

15 $$\left.\begin{array}{c}{LCL}_{i}=\begin{array}{c} {\mu }_{0}-L\sqrt{\frac{{\beta }^{2}{\sigma }_{0}^{2}}{n}} \text{if} i=1\\ {\mu }_{0}-L\sqrt{\frac{{\beta }^{2}{\sigma }_{0}^{2}}{n}+{\left(1-\beta \right)}^{2}\frac{{\sigma }_{0}^{2}}{n\left(i-1\right)} }, \text{if} i>1\end{array}\\ CL={\mu }_{0}\\ {UCL}_{i}=\begin{array}{c} {\mu }_{0}+L\sqrt{\frac{{\beta }^{2}{\sigma }_{0}^{2}}{n}} \text{if} i=1\\ {\mu }_{0}+L\sqrt{\frac{{\beta }^{2}{\sigma }_{0}^{2}}{n}+{(1-\beta )}^{2}\frac{{\sigma }_{0}^{2}}{n(i-1)} }, \text{if} i>1\end{array}\end{array}\right]$$

where $L$ is the coefficient of the control limits and its value depends on the ${ARL}_{o}$.

### Design structure of the proposed NPSR-DH control chart

The newly proposed NPSR-DH control chart is based on the WSR test, an alternative of one sample t-test for distribution-free data which means the assumption of normality or particular distribution is not required. The plotting statistic of the NPSR-DH chart based on the statistic of the WSR test is as follows:

16 $${DH}_{t}={\beta }^{2}{H}_{t}+(1-{\beta }^{2}){\overline{WR} }_{t-1 },$$

The mean of the previous $\left(t-1\right)$ statistics of $WR$ is ${\overline{WR} }_{t-1}=\frac{{\sum }_{i=1}^{t-1}{WR}_{i}}{t-1}$ and the initial value of ${\overline{WR} }_{o}=0$. If $\beta$ sets equal to one in (14) then it turns to Shewhart type WSR chart of Bakir^22^. The mean and variance of ${DH}_{t}$ can be obtained as follows:

$$E\left({DH}_{t}\right)={\beta }^{2}E\left({H}_{t}\right)+\left(\frac{1-{\beta }^{2}}{t-1}\right)\sum_{i=1}^{t-1}E\left({WR}_{i}\right)$$

$$E\left({DH}_{t}\right)=0$$

and

$$Var\left({DH}_{t}\right)={\beta }^{4}Var\left({H}_{t}\right)+{\left(\frac{1-{\tau }^{2}}{t-1}\right)}^{2}{\sum }_{i=1}^{t-1}Var\left({WR}_{i}\right)$$

$$Var\left({DH}_{t}\right)=\left\{\begin{array}{ll}{\beta }^{4}\left(\frac{n\left(n+1\right)\left(2n+1\right)}{6}\right) & \quad \text{for}\,t=1\\ \frac{n\left(n+1\right)\left(2n+1\right)}{6}\left({\beta }^{4}+\frac{{\left(1-{\beta }^{2}\right)}^{2}}{t-1}\right) & \quad \text{for}\,t>1\end{array}\right.$$

The limits of the NPSR-DH chart based on the aforementioned $E\left({DH}_{t}\right)$ and $Var\left({DH}_{t}\right)$ are described as:

17 $$\begin{gathered} UCL = \left\{ {\begin{array}{*{20}l} {T\sqrt {~\beta ^{4} \left( {\frac{{n\left( {n + 1} \right)\left( {2n + 1} \right)}}{6}} \right)} } \hfill & {{\text{for}}\;~t = 1} \hfill \\ {T\sqrt {\frac{{n\left( {n + 1} \right)\left( {2n + 1} \right)}}{6}\left( {\beta ^{4} + \frac{{\left( {1 - \beta ^{2} } \right)^{2} }}{{t - 1}}} \right)} } \hfill & {{\text{for}}\;t > 1} \hfill \\ \end{array} } \right. \hfill \\ CL = 0 \hfill \\ LCL = \left\{ {\begin{array}{*{20}l} { - T\sqrt {~\beta ^{4} \left( {\frac{{n\left( {n + 1} \right)\left( {2n + 1} \right)}}{6}} \right)} ~} \hfill & {{\text{for}}\;~t = 1} \hfill \\ { - T\sqrt {\frac{{n\left( {n + 1} \right)\left( {2n + 1} \right)}}{6}\left( {\beta ^{4} + \frac{{\left( {1 - \beta ^{2} } \right)^{2} }}{{t - 1}}} \right)} } \hfill & {{\text{for}}\;t > 1} \hfill \\ \end{array} } \right. \hfill \\ \end{gathered}$$

where $T$ indicates the coefficient of control limit to determine its width according to the pre-defined IC ARL i.e., ${ARL}_{o}$. The statistic ${DH}_{t}$ is plotted against the limits given in (17). If any value of ${DH}_{t}$ is plotted beyond the limits given in (17), the process is seemed to be OOC, else, it is considered to be IC.

## Performance evaluation

**Figure 1:**
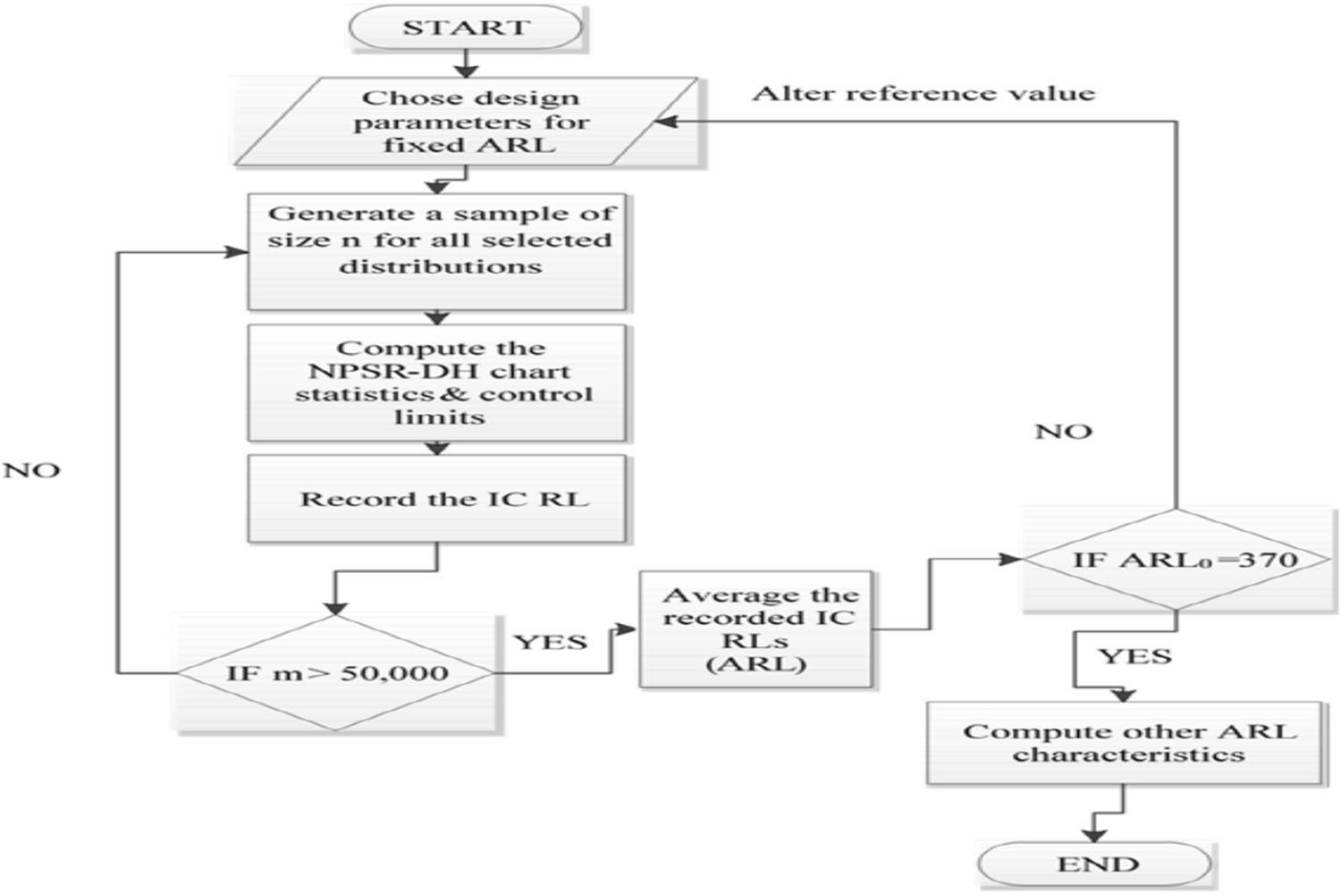
The flow chart based compuatational algorithm of the NPSR_DH chart.

In literature, several statistical measures are available to judge the performance of control schemes. Some of them are for a single value of shift while others for a range of shifts. The *ARL* is the one used more often. There are two types of $ARL$ that is ${ARL}_{o}$ and ${ARL}_{1}$ are used to assess the performance of the control chart. The ${ARL}_{o}$ is the expected number of samples before an OOC point is detected when the process is IC while ${ARL}_{1}$ is the expected number of samples before an OOC signal is received when the process is shifted to an OOC state. A chart is considered more effective as compared to other charts if it has a minimum value of ${ARL}_{1}$ for the same amount of shift i.e., $\delta$. We have also assessed the performance of the NPSR-DH chart by using the other measures of the RL such as (standard deviation of the RL (SDRL), a median of the RL (MDRL), and some percentile points of the RL (${PRL}_{25}, {PRL}_{50} \& {PRL}_{75}$)) because of the skewed behavior of the RL distribution (cf. Naveed et al.^34^, Bataineh et al.^35^ and Shafqat et al.^36^). The RL measures are calculated through Monte Carlo simulations in R language. The ${ARL}_{o}$ of the newly developed NPSR-DH chart depends on the values of $n, T and \beta ,$ i.e., ${ARL}_{o}=f\left(n, \beta , T\right).$ The computational algorithm of the NPSR-DH chart in the form of the flow chart is presented in Fig. 1.

The values of T of the NPSR-DH chart for several choices of $\upbeta$ and sample size $\left(n\right)$ for ${ARL}_{0}\approx 370 \& 500$ are reported in Table 1. It is noted that the values of are increased as $\upbeta$ increases when ${ARL}_{0}\approx 370 \& 500$ (cf. Table 1). It is also noted that for fixed $\upbeta$ the value of $T$ also increases as $n$ is increased (for instance $\upbeta =0.20, n=10, T=2.31,$ $\upbeta =0.20, n=15, T=2.37$, $\text{and}\upbeta =0.20, n=20, T=2.4$, when ${ARL}_{O}=500$ (cf. Table 1).

**Table 1:** The values of $T$ based on various choices of $n\boldsymbol{ }\text{and}\boldsymbol{ }\beta$ attain the desired nominal ${ARL}_{{\varvec{o}}}\approx 370,\boldsymbol{ }500$ for the NPSN-DH chart.

$\left(n, \beta \right)$	${ARL}_{o}\approx 370$		${ARL}_{o}\approx 500$	
	*T*	${ARL}_{o}$	$T$	${ARL}_{o}$
(10, 0.05)	1.3300	369.35	1.3800	499.59
(10, 0.10)	1.6000	371.56	1.6800	500.49
(10, 0.20)	2.1800	369.10	2.3100	501.20
(15, 0.05)	1.3264	368.64	1.3800	502.28
(15, 0.10)	1.6100	370.13	1.7000	501.23
(15, 0.20)	2.2300	371.10	2.3700	499.41
(20, 0.05)	1.3550	371.16	1.3800	498.11
(20, 0.10)	1.6200	371.18	1.7000	499.26
(20, 0.20)	2.2440	369.52	2.4000	501.16

### Normal and non-normal environments

Both the normal and non-normal environments are used to evaluate the performance of the proposed chart. The following distributions are employed to assess the performance of the proposed NPSR-DH chart: (a) A bell-shaped, symmetrical normal distribution denoted by N(0,1), with mean and variance; (b) Platykurtic-shaped symmetrical Student's t-distribution with 4 degrees of freedom and a heavy tail; (c) Laplace distribution, also known as the double exponential distribution, denoted as Lap(0, 1/$\sqrt{2}$); (d) Logistic distribution represented as Log(0,$\sqrt{3}$/π); and (e)Contaminated normal distribution with 5% level of contamination CN(0.05) used to assess the behavior of the proposed chart in the presence of outliers. All the distributions under consideration are adjusted with zero mean and unit standard deviation for valid comparisons. The density functions these distributions are detailed in Table 2.

**Table 2:** Density functions of the continuous distributions under consideration.

(1) Standard normal; $f\left(V\right)=\frac{{e}^{-\frac{{V}^{2}}{2}}}{\sqrt{2\pi }}$, where;$V\in \mathcal{R},{T}_{0}=0 and {\sigma }^{2}=1$
(2) Student’s $t\left(\nu \right); f\left(V\right)=\frac{\Gamma (\frac{u+1}{2})}{\Gamma (\frac{u}{2})\sqrt{u\pi }}{\left(1+\frac{{V}^{2}}{u}\right)}^{\frac{u+1}{2}}$, where; $V\in \mathcal{R},{T}_{0}=0 and {\sigma }^{2}=\frac{u}{u+2}$ and $u=4$
(3) Logistic; $$f\left(V\right)=\frac{{e}^{-\frac{\pi V}{\sqrt{3}}}}{\frac{\sqrt{3}}{\pi }{\left(1+{e}^{-\frac{\pi V}{\sqrt{3}}}\right)}^{2}}$$, where;$$V\in \mathcal{R},{T}_{0}=0 and {\sigma }^{2}=\frac{3}{{\pi }^{2}}$$
(4) Laplace; $$f\left(V\right)=\frac{1}{2}{e}^{-\left|V\right|}$$, where;$$V\in \mathcal{R},{T}_{0}=0 and {\sigma }^{2}=\frac{1}{2}$$
(5) Contaminated Normal (CN); $f\left(V\right)=\frac{0.95{e}^{-\frac{{V}^{2}}{2}}}{\sqrt{2\pi }}+\frac{{0.05e}^{-\frac{{V}^{2}}{2{\sigma }_{0}^{2}}}}{{\sigma }_{0}^{2}\sqrt{2\pi }}$, where;$V\in \mathcal{R},{T}_{0}=0 and {\sigma }^{2}=0.95+0.05{\sigma }_{0}^{2}$

The RL characteristics of the newly developed NPSR-DH chart for N(0,1), t(4), t(8), Lap(0,1/$\sqrt{2}$), Log $(0, \sqrt{3}/\pi )$, and 5% contaminated normal i.e., CN(0.05) under $\upbeta =0.10, n=10 \& T=1.68$ at ${ARL}_{o}\approx 500$ are reported in Table 2. The ${ARL}_{o}$ values of the NPSR-DH chart are identical for all distributions under consideration (cf. Table 2). Furthermore, the distribution of the ${ARL}_{o}$ is positively skewed for all distributions i.e., ${ARL}_{o}>{PRL}_{50}$ (cf. Table 2). The ${ARL}_{1}$ performance of the NPSR-DH chart is relatively better in the case of a Laplace(0,1/$\sqrt{2}$) distribution against other distributions. For instance when $\delta =0.1, 0.25$, the corresponding ${ARL}_{1}$ values for Lap(0,1/$\sqrt{2}$), N(0,1), t(4), t(8), Log $(0, \sqrt{3}/\pi )$, and CN(0.05) distributions are (16.95, 4.83), (24.94, 6.57), (18.31, 4.95), (21.97, 5.85), (22.10, 5.87), and (20.69, 5.51), respectively (cf. Table 2). Moreover, the ${ARL}_{1}$ performance of the NPSR-DH chart for the Log $(0, \sqrt{3}/\pi )$ distribution is comparatively worst against all other distributions (cf. Table 3).

**Table 3:** The run length characteristics of the NPSR-DH chart (${ARL}_{o} \& {SDRL}_{o}$ is allocated in first row and ${PRL}_{25}, {PRL}_{50} \& {PRL}_{75}$ is reported in second row) for $\beta =0.10, n=10 and T=1.68 at {ARL}_{o}\approx 500.$

Distributions	δ									
	0	0.1	0.25	0.5	0.75	1	1.5	2	2.5	3
Normal (0,1)	501.40(903.98) (7, 57, 589.25)	24.94(30.40)	6.57(5.47)	2.70(1.77)	1.68(1.08)	1.22(0.64)	1.01(0.13)	1(0)	1(0)	1(0)
		(5, 13, 34)	(3, 5, 9)	(1, 3, 4)	(1, 1, 3)	(1, 1, 1)	(1, 1, 1)	(1, 1, 1)	(1, 1, 1)	(1, 1, 1)
t(4)		18.31(20.66)	4.95(3.79)	2.22(1.44)	1.48(0.91)	1.15(0.53)	1.02(0.19)	1.00(0.07)	1.00(0.02)	1(0)
		(4, 11, 25)	(3, 4, 7)	(1, 1, 3)	(1, 1, 1)	(1, 1, 1)	(1, 1, 1)	(1, 1, 1)	(1, 1, 1)	(1, 1, 1)
t(8)		21.97(25.31)	5.85(4.73)	2.53(1.64)	1.59(1.01)	1.20(0.61)	1.01(0.16)	1.00(0.04)	1(0)	1(0)
		(5, 12, 30)	(3, 5, 8)	(1, 3, 4)	(1, 1, 3)	(1, 1, 1)	(1, 1, 1)	(1, 1, 1)	(1, 1, 1)	(1, 1, 1)
Laplace(0,1/$\sqrt{2}$)		16.95(18.71)	4.83(3.67)	2.26(1.48)	1.51(0.94)	1.19(0.60)	1.02(0.22)	1.00(0.05)	1.00(0.02)	1(0)
		(4,10,23)	(3, 4, 7)	(1, 1, 3)	(1, 1, 1)	(1, 1, 1)	(1, 1, 1)	(1, 1, 1)	(1, 1, 1)	(1, 1, 1)
Logistic $(0, \sqrt{3}/\pi )$		22.10(26.24)	5.87(4.70)	2.55(1.67)	1.58(0.99)	1.21(0.62)	1.01`(0.17)	1(0.04)	1(0)	1(0)
		(4, 12, 30)	(3, 5, 8)	(1, 3, 4)	(1, 1, 3)	(1, 1, 1)	(1, 1, 1)	(1, 1, 1)	(1, 1, 1)	(1, 1, 1)
CN(0.05)		20.69(23.38)	5.51(4.37)	2.39(1.55)	1.49(0.92)	1.17(0.56)	1.01(0.17)	1(0.06)	1(0.05)	1(0.02)
		(4, 12, 29)	(3, 4, 7)	(1, 3, 3)	(1, 1, 1)	(1, 1, 1)	(1, 1, 1)	(1, 1, 1)	(1, 1, 1)	(1, 1, 1)

### Distributional comparisons based on ${{\varvec{A}}{\varvec{R}}{\varvec{L}}}_{1}$ values

A comprehensive comparison of the newly developed control chart versus existing counterparts is done, in this section. Also, the plotting statistic and control limits of the existing and proposed charts are reported in Table 1. The efficiency comparisons of the NPSR-DH chart with the SEWMA chart, SHWMA chart, NPSN- E chart, NPSR-E chart, and NPSR-H chart are made on the basis of ARL, SDRL, and MDRL values for all selected distributions, and results are reported in Tables 4, 5, 6, 7, 8.

**Table 4:** The OOC run length characteristics of the proposed and existing charts for $\beta =0.05 and n=10 at {ARL}_{o}\approx 500$ under $N(0, 1).$

Control charts	Characteristics	δ									EQL
		0.1	0.25	0.5	0.75	1	1.5	2	2.5	3	
SEWMA with L = 2.613	ARL	57.6	15.4	6.7	4.4	3.3	2.3	2	1.7	1.1	5.62
	SDRL	42.3	6.6	1.9	1.1	0.6	0.4	0.1	0.5	0.3	
	MDRL	46	14	6	4	3	2	2	2	1	
SHWMA with L = 2.608	ARL	51.6	11.8	4.1	2.4	1.5	1	1	1	1	3.29
	SDRL	36.8	7.6	2.1	1.3	0.9	0.2	0	0	0	
	MDRL	44	10	4	3	1	1	1	1	1	
NPSN-E with L = 2.612	ARL	83.5	21	9	6	4.8	3.7	3.1	3	3	10.02
	SDRL	66.5	10.6	2.8	1.4	0.8	0.6	0.4	0.1	0	
	MDRL	64	19	9	6	5	4	3	3	3	
NPSR-E with L = 2.610	ARL	63.3	16.8	7.6	5.4	4.5	4	4	4	4	12.40
	SDRL	47.1	7.3	2	1	0.6	0.1	0	0	0	
	MDRL	50	15	7	5	4	4	4	4	4	
NPSR-H with T = 2.430	ARL	49.4	11.8	4.4	2.8	2	1.2	1	1	1	3.45
	SDRL	37.2	7.8	2.1	1.2	1	0.6	0.2	0	0	
	MDRL	42	10	4	3	2	1	1	1	1	
NPSR-DH with T = 1.38	ARL	15.4	5	2.3	1.5	1.1	1	1	1	1	3.12
	SDRL	21.4	4.3	1.5	0.9	0.5	0.1	0	0	0	
	MDRL	7	4	1	1	1	1	1	1	1

**Table 5:** The OOC run length characteristics of the proposed and existing charts for $\beta =0.05 and n=10 at {ARL}_{o}\approx 500$ under $t (4).$

Control charts	Characteristics	δ									EQL
		0.1	0.25	0.5	0.75	1	1.5	2	2.5	3	
SEWMA with L = 2.613	ARL	59.4	15.4	6.8	4.4	3.3	2.3	2	1.7	1.1	5.64
	SDRL	44.3	6.7	1.9	1	0.6	0.4	0.2	0.5	0.3	
	MDRL	47	14	7	4	3	2	2	2	1	
SHWMA with L = 2.608	ARL	64.4	14.4	4.8	2.7	1.8	1.1	1	1	1	3.41
	SDRL	41.9	8.4	2.2	1.3	1	0.3	0.1	0	0	
	MDRL	57	13	4	3	1	1	1	1	1	
NPSN-E with L = 2.612	ARL	53.7	14.5	7	5	4.2	3.5	3.2	3	3	9.81
	SDRL	38	5.9	1.8	0.9	0.7	0.5	0.4	0.2	0.1	
	MDRL	43	13	7	5	4	3	3	3	3	
NPSR-E with L = 2.610	ARL	46.3	13.1	6.5	4.9	4.3	4	4	4	4	12.31
	SDRL	31.5	5	1.5	0.8	0.5	0.1	0	0	0	
	MDRL	38	12	6	5	4	4	4	4	4	
NPSR-H with T = 2.430	ARL	44.3	8.7	3.5	2.3	1.8	1.2	1.1	1	1	3.44
	SDRL	31.5	5.4	1.7	1.2	1	0.6	0.4	0.2	0.1	
	MDRL	38	7	3	3	1	1	1	1	1	
NPSR-DH with T = 1.38	ARL	12	4	1.9	1.3	1.1	1	1	1	1	3.09
	SDRL	14.9	3.1	1.3	0.7	0.4	0.1	0	0	0	
	MDRL	6	3	1	1	1	1	1	1	1

**Table 6:** The OOC run length characteristics of the proposed and existing charts for $\beta =0.05 and n=10 at {ARL}_{o}\approx 500$ under $Lap (0, 1/\sqrt{2}).$

Control charts	Characteristics	δ									EQL
		0.1	0.25	0.5	0.75	1	1.5	2	2.5	3	
SEWMA with L = 2.613	ARL	59.3	15.3	6.7	4.4	3.3	2.3	2	1.7	1.1	5.64
	SDRL	44.2	6.6	1.9	1	0.6	0.4	0.1	0.5	0.3	
	MDRL	47	14	6	4	3	2	2	2	1	
SHWMA with L = 2.608	ARL	56.1	12.8	4.4	2.5	1.6	1	1	1	1	3.32
	SDRL	38.1	7.9	2.2	1.3	0.9	0.3	0	0	0	
	MDRL	49	11	4	3	1	1	1	1	1	
NPSN-E with L = 2.612	ARL	38	12.2	6.6	5	4.3	3.6	3.2	3.1	3	9.94
	SDRL	24.2	4.6	1.6	0.9	0.7	0.6	0.4	0.3	0.1	
	MDRL	32	11	6	5	4	4	3	3	3	
NPSR-E with L = 2.610	ARL	41.8	12.7	6.5	5	4.3	4	4	4	4	12.31
	SDRL	27.5	1.7	1.5	0.8	0.5	0.1	0	0	0	
	MDRL	35	12	6	5	4	4	4	4	4	
NPSR-H with T = 2.430	ARL	32.9	8.2	3.6	2.4	1.8	1.3	1.1	1	1	3.47
	SDRL	24.4	5	1.7	1.2	1	0.6	0.3	0.2	0.1	
	MDRL	28	7	3	3	1	1	1	1	1	
NPSR-DH with T = 1.38	ARL	10.9	3.9	1.9	1.3	1.1	1	1	1	1	3.09
	SDRL	13.3	3	1.3	0.8	0.4	0.1	0	0	0	
	MDRL	6	3	1	1	1	1	1	1	1

**Table 7:** The OOC run length characteristics of the proposed and existing charts for $\beta =0.05 and n=10 at {ARL}_{o}\approx 500$ under $Log (0, \sqrt{3}/\pi ).$

Control charts	Characteristics	δ									EQL
		0.1	0.25	0.5	0.75	1	1.5	2	2.5	3	
SEWMA with L = 2.613	ARL	58.8	15.4	6.7	4.4	3.3	2.3	2	1.7	1.1	5.63
	SDRL	43.6	6.8	1.9	1	0.6	0.4	0.1	0.5	0.3	
	MDRL	46	14	6	4	3	2	2	2	1	
SHWMA with L = 2.608	ARL	53.5	12.4	4.2	2.4	1.6	1	1	1	1	3.31
	SDRL	37.2	7.7	2.1	1.3	0.9	0.2	0	0	0	
	MDRL	46	11	4	3	1	1	1	1	1	
NPSN-E with L = 2.612	ARL	68.1	17.7	8	5.6	4.5	3.6	3.2	3	3	9.95
	SDRL	52.5	8.1	2.3	1.2	0.8	0.6	0.4	0.2	0.1	
	MDRL	53	16	8	5	4	4	3	3	3	
NPSR-E with L = 2.610	ARL	57.1	15.3	7.2	5.2	4.4	4	4	4	4	12.36
	SDRL	41.4	6.4	1.8	0.9	0.6	0.1	0	0	0	
	MDRL	45	14	7	5	4	4	4	4	4	
NPSR-H with T = 2.430	ARL	43.9	10.4	4	2.6	1.9	1.2	1	1	1	3.41
	SDRL	32.9	6.8	2	1.2	1	0.6	0.3	0.1	0	
	MDRL	37	9	4	3	1	1	1	1	1	
NPSR-DH with T = 1.38	ARL	13.9	4.6	2.1	1.4	1.1	1	1	1	1	3.11
	SDRL	18.2	3.9	1.4	0.8	0.5	0.1	0	0	0	
	MDRL	7	4	1	1	1	1	1	1	1

**Table 8:** The OOC run length characteristics of the proposed and existing charts for $\beta =0.05 and n=10 at {ARL}_{o}\approx 500$ under $CN$ with 5% contamination.

Control charts	Characteristics	δ									EQL
		0.1	0.25	0.5	0.75	1	1.5	2	2.5	3	
SEWMA with L = 2.613	ARL	56.5	14.9	6.6	4.3	3.3	2.2	2	1.6	1.1	5.49
	SDRL	41.5	6.3	1.8	1	0.6	0.4	0.2	0.5	0.3	
	MDRL	45	14	6	4	3	2	2	1	1	
SHWMA with L = 2.608	ARL	50.5	11.5	4	2.3	1.5	1	1	1	1	3.28
	SDRL	35.7	7.3	2.1	1.2	0.9	0.2	0	0	0	
	MDRL	44	10	4	3	1	1	1	1	1	
NPSN-E with L = 2.612	ARL	75.8	19.5	8.6	5.9	4.7	3.6	3.1	3	3	9.95
	SDRL	59.3	9.2	2.6	1.3	0.8	0.6	0.3	0.1	0	
	MDRL	59	18	8	6	5	4	3	3	3	
NPSR-E with L = 2.610	ARL	60.7	16.2	7.4	5.3	4.4	4	4	4	4	12.38
	SDRL	44.6	6.9	1.9	0.9	0.6	0.1	0	0	0	
	MDRL	48	15	7	5	4	4	4	4	4	
NPSR-H with T = 2.430	ARL	46.2	11.1	4.2	2.7	2	1.2	1	1	1	3.44
	SDRL	35.4	7.1	2	1.2	1	0.5	0.2	0	0	
	MDRL	39	10	4	3	2	1	1	1	1	
NPSR-DH with T = 1.38	ARL	13.7	4.4	2	1.3	1.1	1	1	1	1	3.10
	SDRL	17.8	3.6	1.4	0.8	0.4	0.1	0.1	0	0	
	MDRL	7	4	1	1	1	1	1	1	1	

A measure called a percentage decrease in $ARL ({PD}_{ARL})$ is also used for comparison purpose and mathematically, it is defined as:

18 $${PD}_{ARL}=\left(\frac{{ARL}_{0}-{ARL}_{1}}{{ARL}_{0}}\right)\times 100\%$$

A control chart with a larger ${PD}_{ARL}$ value is considered to be efficient.

The ${PD}_{ARL}$ is just used to compare the performance of the control charts for specific shifts and not use for assessing the overall performance of the control charts. So to evaluate the overall performance of the proposed and existing charts, a well-known overall performance measure called the extra quadratic loss ($EQL$) suggested by Zhang and Wu^37^ is also utilized in this study. The $EQL$ is the weighted average of ${ARL}_{1}$ values over a range of shifts $\left(\delta \right)$ by taking into account ${\delta }^{2}$ as weight. Mathematically, the $EQL$ is defined as:

19 $$EQL={\left({\delta }_{max}-{\delta }_{min}\right)}^{-1}\underset{{\delta }_{min}}{\overset{{\delta }_{max}}{\int }} {\delta }^{2}{ARL}_{1}\left(\delta \right)d\delta$$

A control chart can be considered best among others if it shows minimum value of $EQL$ provided that all control charts have same ${ARL}_{0}$ values.

The distributional comparisons between proposed and existing charts are given below:

#### Standard normal distribution

The proposed NPSR-DH chart offers better performance than the SEWMA, SHWMA, NPSN-E, NPSR-E, and NPSR-H charts against the standard normal distribution. For instance when $\delta =0.1, 0.25, 0.75$, the corresponding ${ARL}_{1}$ values of NPSR-DH, SEWMA, SHWMA, NPSN-E, NPSR-E, and NPSR-H charts are (15.4, 5.0, 1.5), (57.6, 15.4, 4.4), (51.6, 11.8, 4.1), (83.5, 21.0, 6.0), (63.3, 16.8, 5.4), and (49.4, 11.8, 2.4), respectively (cf. Table 4). Moreover, at $\delta =0.1$, the ${PD}_{ARL}$ in NPSR-DH, SEWMA, SHWMA, NPSN-E, NPSR-E, and NPSR-H charts are 96.92%, 88.48%, 89.68%, 83.3%, 87.3%, and 90.12%, respectively.

#### t(4) distribution

The proposed NPSR-DH chart shows relatively improved performance than the SEWMA, SHWMA, NPSN-E, NPSR-E, and NPSR-H charts against the t(4) distribution. For instance when $\delta =0.1, 0.25, 0.75$, the corresponding ${ARL}_{1}$ values of NPSR-DH, SEWMA, SHWMA, NPSN-E, NPSR-E, and NPSR-H charts are (12.0, 4.0, 1.3), (59.4, 15.4, 4.4), (64.4, 14.4, 4.0), (53.7, 14.5, 5.0), (46.3, 13.1, 5.0), and (44.3, 8.7, 2.3), respectively (cf. Table 5). Also, at $\delta =0.1$, the ${PD}_{ARL}$ in NPSR-DH, SEWMA, SHWMA, NPSN-E, NPSR-E, and NPSR-H charts are 97.6%, 88.12%, 87.12%, 89.26%, 90.74%, and 91.14%, respectively.

#### Laplace distribution

The proposed NPSR-DH chart indicates better ${ARL}_{1}$ performance than the SEWMA, SHWMA, NPSN-E, NPSR-E, and NPSR-H charts against the Laplace distribution. For instance when $\delta =0.1, 0.25, 0.75$, the corresponding ${ARL}_{1}$ values of NPSR-DH, SEWMA, SHWMA, NPSN-E, NPSR-E, and NPSR-H charts are (10.9, 3.9, 1.3), (59.3, 15.3, 4.4), (56.1, 12.8, 2.5), (38.0, 12.2, 5.0), (41.8, 12.7, 5.0), and (32.9, 8.2, 2.4), respectively (cf. Table 6). Furthermore, at $\delta =0.25$, the ${PD}_{ARL}$ in NPSR-DH, SEWMA, SHWMA, NPSN-E, NPSR-E, and NPSR-H charts are 99.22%, 96.94%, 97.44%, 97.56%, 97.46%, and 98.36%, respectively.

#### Logistic distribution

The proposed NPSR-DH chart shows enhanced performance than the SEWMA, SHWMA, NPSN-E, NPSR-E, and NPSR-H charts against the Logistic distribution. For instance when $\delta =0.1, 0.25, 0.75$, the corresponding ${ARL}_{1}$ values of NPSR-DH, SEWMA, SHWMA, NPSN-E, NPSR-E, and NPSR-H charts are (13.9, 4.6, 2.1), (58.8, 15.4, 4.4), (53.5, 12.4, 2.4), (68.1, 17.7, 5.6), (57.1, 15.3, 5.2), and (43.9, 10.4, 5.0), respectively (cf. Table 7). Additionally, at $\delta =0.25$, the ${PD}_{ARL}$ in NPSR-DH, SEWMA, SHWMA, NPSN-E, NPSR-E, and NPSR-H charts are 99.08%, 96.92%, 97.52%, 97.46%, 96.94%, and 97.92%, respectively.

#### Contaminated normal distribution

The proposed NPSR-DH chart offers relatively superior performance than the SEWMA, SHWMA, NPSN-E, NPSR-E, and NPSR-H charts against the contaminated normal distribution with 5% contamination. For instance when $\delta =0.1, 0.25, 0.75$, the corresponding ${ARL}_{1}$ values of NPSR-DH, SEWMA, SHWMA, NPSN-E, NPSR-E, and NPSR-H charts are (13.7, 4.4, 1.3), (56.5, 14.9, 4.3), (50.5, 11.5, 2.3), (75.8, 19.5, 5.9), (60.7, 16.2, 5.3), and (46.2, 11.1, 2.7), respectively (cf. Table 8). Also, at $\delta =0.1$, the ${PD}_{ARL}$ in NPSR-DH, SEWMA, SHWMA, NPSN-E, NPSR-E, and NPSR-H charts are 97.26%, 88.7%, 89.9%, 84.84%, 87.86%, and 90.76%, respectively.

### Distributional comparisons based on SDRL values

The proposed NPSR-DH chart offers quite a stable SDRL performance against the SEWMA, SHWMA, NPSN-E, NPSR-E, and NPSR-H charts for all selected distributions. For instance when $\delta =0.1,$ the corresponding $SDRL$ values of NPSR-DH, SEWMA, SHWMA, NPSN-E, NPSR-E, and NPSR-H charts for (standard normal, t(4), Laplace, logistic, contaminated normal distributions) are (21.4, 14.9, 13.3, 18.2, 17.8), (42.3, 44.3, 44.2, 43.6, 41.5), (36.8, 41.9, 38.1, 37.2, 35.7), (66.5, 38.0, 24.2, 52.5, 59.3), (47.1, 31.5, 27.5, 41.4, 44.6), and (37.2, 31.5, 24.4, 32.9, 35.4), respectively (cf. Tables 4, 5, 6, 7, 8).

### Distributional comparisons based on MDRL values

The proposed NPSR-DH chart offers smaller MDRL values against the SEWMA, SHWMA, NPSN-E, NPSR-E, and NPSR-H charts for all selected distributions. For instance when $\delta =0.1,$ the corresponding $MDRL$ values of NPSR-DH, SEWMA, SHWMA, NPSN-E, NPSR-E, and NPSR-H charts for (standard normal, t(4), Laplace, logistic, contaminated normal distributions) are (7.0, 6.0, 6.0, 7.0, 7.0), (46.0, 47.0, 47.0, 46.0, 45.0), (44.0, 57.0, 49.0, 46.0, 44.0), (64.0, 43.0, 53.0, 52.5, 59.0), (50.0, 38.0, 35.0, 45.0, 48.0), and (42.0, 38.0, 28.0, 37.0, 39.0), respectively (cf. Tables 4, 5, 6, 7, 8).

### Graphical comparisons based on ${{\varvec{A}}{\varvec{R}}{\varvec{L}}}_{1}$ values

**Figure 2:**
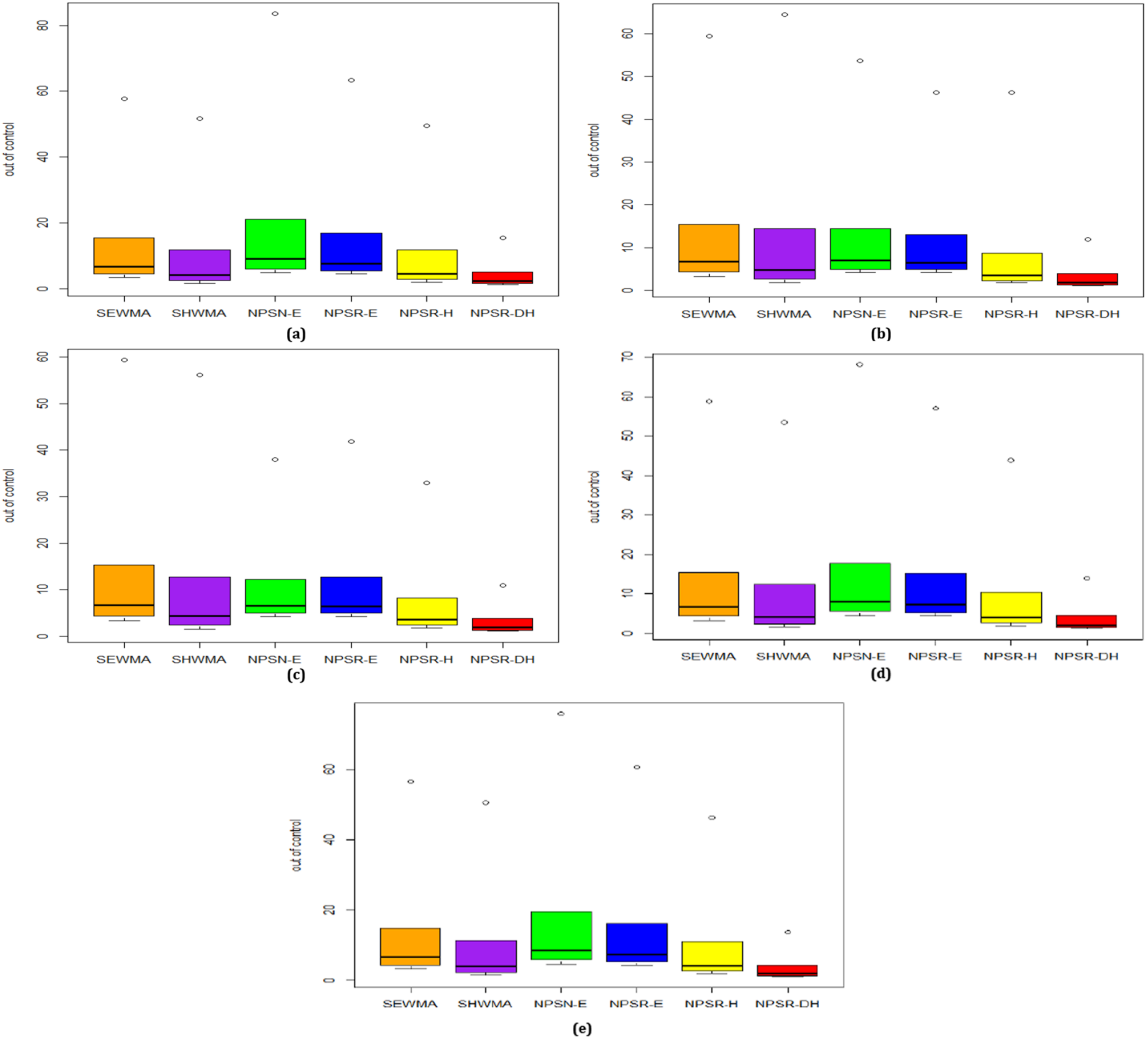
Boxplot based comparison between proposed and existing chart under (**a)** Normal (**b)** t(4) (**c)** Laplace (**d)** Logistic (**e)** Contaminated normal.

The boxplot based graphical comparisons between the proposed NPSR-DH and existing charts constructed on the ${ARL}_{1}$ values are presented in Fig. 2a–e against all selected distributions. The following charts are presenting the inferior performance for the selected distributions such as: NPSN-E chart for the standard normal distribution (cf. Fig. 2a), SHWMA chart for t(4) distribution, (cf. Fig. 2b); SEWMA for Laplace distribution(cf. Fig. 2c), SEWMA for Laplace distribution(cf. Fig. 2c), and NPSN-E for both logistic and contaminated normal distribution (cf. Fig. 2d–e). Moreover, the proposed NPSR-DH chart takes an edge over all the other charts investigated in this study for all selected choices of distributions (cf. Fig. 2a–e).

### Overall distributional comparisons based on EQL

The overall distributional comparisons between proposed NPSR-DH and SEWMA, SHWMA, NPSN-E, NPSR-E, and NPSR-H charts are done on the basis of the values of EQL. It is clearly revealed from Tables 4, 5, 6, 7, 8 that the proposed NPSR-DH chart is better than the SEWMA, SHWMA, NPSN-E, NPSR-E, and NPSR-H charts in terms of overall performance (for instance the EQL values of the proposed NPSR-DH, SEWMA, SHWMA, NPSN-E, NPSR-E, and NPSR-H are 3.11, 5.63, 3.31, 9.35,12.36, and 3.41, respectively in case of Logistic distribution (cf. Table 7)). Moreover, the overall performance of the NPSR-DH chart is relatively better under Laplace distribution.

From all the above-mentioned comparisons, it concludes that the NPSR-DH chart performs outstandingly well to quickly detect shifts in the process location against the existing charts for all selected distributions.

## Industrial application of the NPSR-DH chart

**Figure 3:**
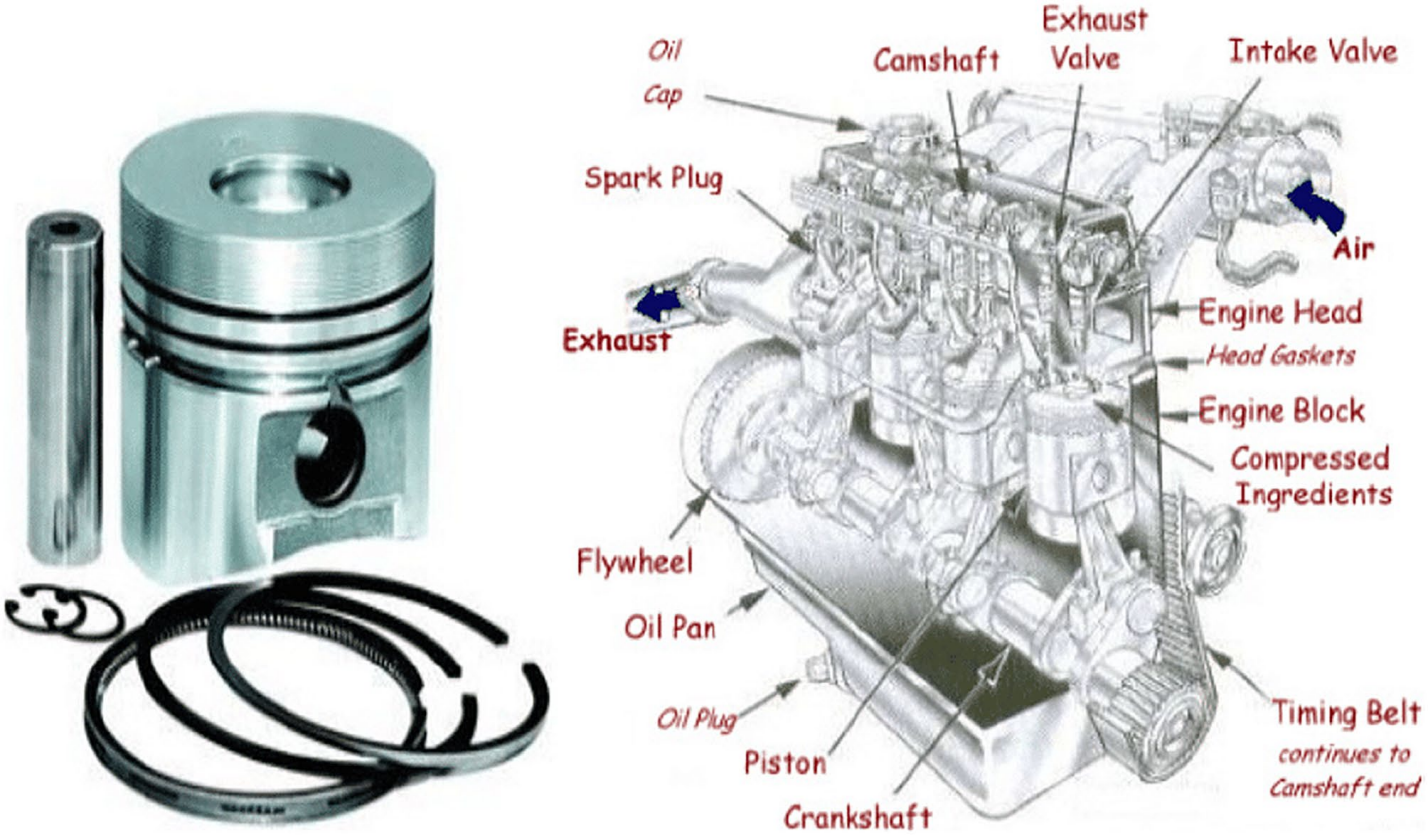
Pictorial description of inside diameter of the automobile piston rings.

In this section, we have presented the real-life industrial application of the NPSR-DH chart. In this industrial process the “inside diameter of the automobile engine piston rings” is considered as the variable of interest (cf. Montgomery^1^). The piston ring is a cap with a spring-like property and it is positioned on the outside diameter of an automobile engine. The main advantage of the piston ring is that its stops the leakage of the boiling gases from the combustion slot. The size of the piston ring diameter of an engine is generally considered to be within the range of 74.000 mm ± 0.05 mm. The pictorial description of the automobile engine piston rings is presented in Fig. 3 (cf. Zafar et al.^38^). Also, the values of the design parameters of the proposed NPSR-DH and existing charts for the piston ring data set is reported in Table 9 when ${ARL}_{o}\approx 500$.

**Table 9:** Values of design parameters of the existing and proposed charts.

Charts	Design parameters
NPSR-E chart	$\beta =0.05, L=2.602$
NPSR-H chart	$\beta =0.05, T=2.0701$
NPSR-DH chart	$\beta =0.05, T=2.322$

**Figure 4:**
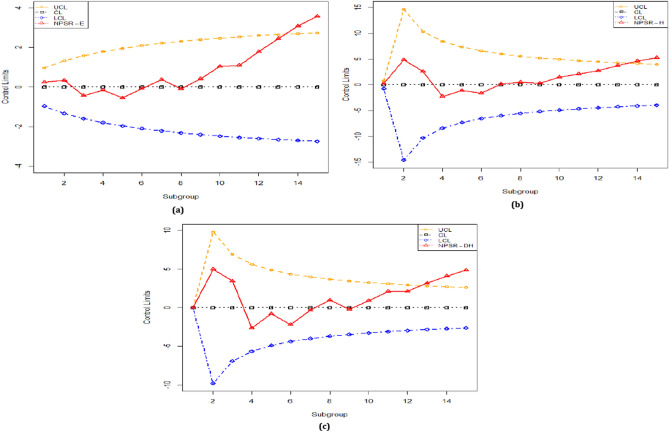
An industrial application based on real-life dataset for (**a)** NPSR-E, (**b)** NPSR-H, (**c)** NPSR-DH charts.

The NPSR-E and NPSR-H charts issue the $\text{OOC}$ signals at the 14th sample (cf. Figure 4a and Fig. 4b). But, the proposed NPSR-DH chart issue the $\text{OOC}$ signal at the 13th (cf. Fig. 4c). So, the proposed NPSR-DH chart has an enhanced shift detection capability than to the existing counterparts and these results also coincide with the results given in Section "Performance evaluation".

## Conclusion and recommendations

Control charts are magnificent statistical monitoring techniques that are commonly applied in industrial and non-industrial processes. For producing and manufacturing high-quality products these industries required highly sensitive monitoring devices which trace deteriorations in the processes effectively. In most of the ongoing processes assumption of normality is hard to meet which leads to parametric monitoring structures invalid comparisons. In this article, a new NPSR-DH scheme has designed based on Wilcoxon signed rank statistic to address small shifts in the process location efficiently. The proposed design proved in control robust for all distributions and more effective for heavy tailed and skewed distributions. The EQL values indicate that the proposed NPSR-DH chart shows superiority against the existing counterpart's charts selected for this study.

The scope of the charting structure explored in this article may also be extended for individual observations based on univariate and multivariate versions. Moreover, the Bayesian aspects of the proposed design structure may also be the potential for future research.

## Data Availability

The datasets used and/or analyzed during the current study are available from the corresponding author on reasonable request.
